# Prevalence of hepatitis B surface antibody among previously vaccinated healthcare workers in Tashkent, Uzbekistan

**DOI:** 10.1080/21645515.2024.2435142

**Published:** 2024-12-18

**Authors:** Rafail Ibragimov, Dilyara Nabirova, Alfiya Denebaeva, Botirjon Kurbanov, Roberta Horth

**Affiliations:** a Central Asia Field Epidemiology Training Program, Almaty, Kazakhstan; b Department of Medical and Preventive Care, Kazakh National Medical University Named After S. D. Asfendiyarov, Almaty, Kazakhstan; c Department of Scientific Research, Innovation, and Training, Committee for Sanitary and Epidemiological Welfare and Public Health under the Ministry of Health, Tashkent, Uzbekistan; d Division of Global Health Protection in Central Asia, United States Centers for Disease Control and Prevention, Almaty, Kazakhstan; e AIDS Prevention and Control Center, Almaty, Kazakhstan

**Keywords:** Hepatitis B virus, immunization, vaccination, health personnel, hepatitis B antibodies, immunization programs, Uzbekistan

## Abstract

Healthcare workers (HCW) have high occupational risk for hepatitis B and Uzbekistan held two HCW vaccination campaigns in 2015 and 2022. Hepatitis B antibody testing (anti-HBs) after Hepatitis B (HepB) vaccination is recommended by the U.S. CDC and WHO for HCW, but Uzbekistan does not have such a policy. In 2023, we randomly selected HCW from the campaign registries. Participants who agreed were interviewed at their workplaces. Vaccination doses were self-reported. Testing for hepatitis B surface antigen (HBsAg), Total hepatitis B core antibody (anti-HBc), and anti-HBs were concurrently performed. We used multivariable Poisson regression to assess factors associated with anti-HBs ≥10 mIU/mL. Of 334 participants, 205 were vaccinated in 2015 and 129 in 2022. Median age was 40 years (interquartile range 35–49 years), and 87% were female. Most (71%) reported having completed the three doses, 21% two doses and 7% one dose. Testing revealed that 5% had an active HBV infection, 4% had a resolved infection, and 91% had detectable vaccine-derived antibodies. Among those (*n* = 303), 71% had anti-HBs ≥10 mIU/mL. For those who reported receiving 1, 2, and 3 doses, protective titers were 59%, 70%, and 72%, respectively. Protective titers were lower for HCW that worked in clinics versus hospitals (aPR = 0.92, CI: 0.87–0.98, *p* = .01) adjusting for age, dose number and presence of chronic conditions. Strategies to improve completion of the 3-dose series and policies for post-vaccination immunity testing 1–2 months after completion of the 3-dose HepB series could help identify workers who may require revaccination or are currently infected.

## Introduction

Viral hepatitis was among the leading causes of death among communicable diseases globally in 2022, and an estimated 254 million people globally are living with hepatitis B.^1^ The hepatitis B virus (HBV) can be transmitted through contact with human blood and infectious bodily fluids. Healthcare workers (HCW) have increased occupational risk of hepatitis B than the general adult population.^2,3^ In the European region, 300,000 hCW are exposed to HBV each year through unintentional percutaneous contact, of which 15,000 become infected.^4^ The risk of contracting HBV among unvaccinated HCW after a single exposure ranges from 6% to 30%.^2,3,5,6^

Vaccination against Hepatitis B is part of a comprehensive plan to prevent and control virus transmission. To reduce the risk of occupational exposures, it is recommended that HCW exposed to blood and blood products receive Hepatitis B vaccination (HepB) following a 0, 1, and 6 month vaccination schedule.^2,7,8^ Vaccinated workers should also receive post-vaccination testing for hepatitis B antibody (anti-HBs) after completion of the 3-dose series to ensure they have developed protective immunity. For workers who do not develop protective immunity and do not have current HBV infection, revaccination is recommended.^8,9^ Anti-HBs ≥10 mIU/mL after vaccination are considered protective against HBV infection.^10^ It is estimated that about 5–10% of healthy people under 40 years old fail to achieve protective levels of immunity after completion of an HBV vaccination series.^11^

In Uzbekistan, a country with population of 35.6 million, there are an estimated 2.5 million people with HBV, of which an estimated 10% are diagnosed, and only 12,500 (5% of those diagnosed) have ever received any treatment for the infection.^12^ In 2015, the country carried out a hepatitis B vaccination campaign among HCW who reported having direct contact with blood as part of their work. In Tashkent, the capital and largest city 13,657 hCW were vaccinated, of whom 11,339 were still employed on January 1, 2022. A second hepatitis B vaccination campaign was held in July 2022 and an additional 6,950 hCW who had not been vaccinated in the 1^st^ campaign in Tashkent were vaccinated.^13^ Both vaccination campaigns provided hepatitis B vaccine (HepB) free of charge to all public healthcare facilities across the country.^14^ Each facility was responsible for vaccinating their eligible workers with 3-doses as indicated by a national presidential decree.^13^ The national schedule for hepatitis B states that doses should be administered at 0-, 1- and 6-months intervals.

Post-vaccination serological testing after completion of the 3-dose series has not been adopted as a policy after hepatitis B vaccination among HCW in Uzbekistan. It was also not performed after vaccination during the 2015 and 2022 campaigns. The proportion of HCW with anti-HBs ≥10 mIU/mL was unknown. Due to the high occupational risk of HBV exposure among HCW, it was important to assess anti-HBs levels in this group. This information can be used to advocate for policy recommendations for post-vaccination testing and revaccination.

## Materials and methods

We conducted a cross-sectional study during June – August 2023 in public medical institutions in Tashkent city. Participants were HCW (physicians, nurses, auxiliary medical staff, and laboratorians) selected using simple random sampling from a database of HCW who received at least one vaccine dose during the hepatitis B vaccination campaigns in 2015 and 2022 (11,339 in 2015 and 6,950 in 2022). HCW who were no longer employed in the public medical system were excluded. The minimum sample size needed for our study was 269, based on a 95% confidence level, and a 5% margin of error around an estimated prevalence of anti-HBs ≥10 mIU/mL of 77%, to account for waning immunity among a population vaccinated nearly 10 years prior.^15^ We increased the sample to 340 participants to account for potential non-response and to have sufficient power to compare proportions by vaccination year.

Selected participants were recruited at their place of work and provided written informed consent. Trained interviewers conducted face-to-face computer-assisted interviews using structured questionnaires that had been programmed in the Kobo Toolbox. The questionnaire included participant demographic and health characteristics. Vaccination dates and doses were self-reported. Thirty-seven HCW (11%) had self-reports cross-checked with their vaccination records which they provided; no discrepancies were identified between self-reports and records. The remaining HCW did not have their vaccination records with them.

Participants consented to venous blood draws of approximately 4 ml. Samples were stored for under 8 hours in temperature monitored refrigerators and transferred daily to the virological laboratory of the National Reference Laboratory of the Committee for Sanitary and Epidemiological Welfare and Public Health under the Ministry of Health of Uzbekistan. At the reference lab, all samples were aliquoted on day of receipt and stored at −10 to −40°C in temperature monitored freezers.

Blood serum was tested for hepatitis B surface antigen (HBsAg), hepatitis B surface antibody (anti-HBs), and total antibody to hepatitis B core antigen (Total anti-HBc) using Vector-Best reagents (Novosibirsk, Russia) in August 2023. Testing was carried out in accordance with the manufacturer’s instructions. HBsAg and total anti-HBc were determined qualitatively by ELISA and anti-HBs quantitatively. Tests were conducted concurrently.

Test results were interpreted as:^16^

- Current HBV infection: Positive HBsAg and positive Total anti-HBc and anti-HBs negative
- Resolved HBV infection: Negative HBsAg and positive total anti-HBc, and anti-HBs positive
- Immune due to vaccination: anti-HBs positive (Protective: anti-HBs ≥10 mIU/mL) and negative HBsAg and negative total anti-HBc
- Susceptible to infection: total anti-HBc negative, HBsAg negative and anti-HBs negative

Only people with immunity due to vaccination were included in subsequent analyses.

Data cleansing and analysis were performed in R version 4.3.1 (The R Foundation, Vienna, Austria). We reported median and interquartile ranges for quantitative variables and proportions and 95% confidence intervals for qualitative variables. Geometric mean titers and a 95% confidence interval were calculated, and the Kruskal-Wallis test was used to determine the difference between the groups. The prevalence ratio was used to determine the association of factors with the presence of an immune response to vaccination.^17^ Multivariable Poisson regression was performed to adjust the prevalence ratio. The model included categorical variables for age, occupation, place of work, vaccine doses and presence of chronic diseases. The results were considered statistically significant when the p-value was less than 0.05.

No personally identifiable information was included in questionnaires or laboratory samples. The study was approved by the Ethics Committee under the Ministry of Health of the Republic of Uzbekistan. Ethical approval of the study was received from the local ethical commission of the NAO Kazakh National Medical University, named after N.N. S.D. Asfendiyarov, Kazakhstan (No. 13 (149), 03/29/2024). This activity was reviewed by the U.S. CDC, deemed not research, and was conducted consistent with applicable federal law and U.S. CDC policy (See e.g., 45 C.F.R. part 46.102(l),^2^ 21 C.F.R. part 56; 42 U.S.C. §241(d); 5 U.S.C. §552a; 44 U.S.C. §3501 et seq.).

## Results

**Figure 1.**
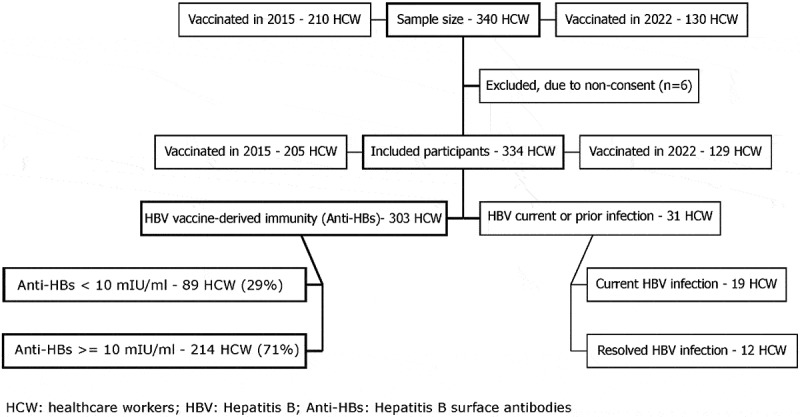
Participant recruitment diagram for a study of immune response after vaccination against hepatitis B in 2015 and 2022, Tashkent, Uzbekistan.

A total of 340 hCW were selected for the study, of whom 6 (2%) did not consent to participate (Figure 1). Of 334 participants included, 205 (61%) had received at least one dose of hepatitis B vaccine (HepB) in the 2015 and 129 (39%) in the 2022 hCW vaccination campaigns. Among participants tested (*n* = 334), 31 were excluded from further analysis due to current (6%; *n* = 19) or resolved HBV infection (4%; *n* = 12). The remaining 303 (91%) had detectable anti-HBs. Among whom, 71% had anti-HBs ≥10 IU/ml.

Among the 334 participants, the median age was 40 years (interquartile range 35–49 years) (Table 1). The median age was 42 years (interquartile range 36–50) for physicians, 38 years (33–46) for laboratorians, 38 years (34–46) for nurses, and 49 years (43–54) for auxiliary staff. Median age did not differ by occupation (not shown in table). Most participants were female (87%) and 52% were nurses. About half (53%) worked in hospitals, with the median length of employment being 15 years (interquartile range 9–22 years). About three-quarters (72%) reported that they completed the three-dose vaccine course.

**Table 1.** Characteristics of healthcare workers vaccinated against HBV in 2015 and 2022 (*n* = 334), Tashkent, Uzbekistan.

Characteristics	Total *n* = 334^a^	Vaccinated in 2015 *n* = 205^a^	Vaccinated in 2022 *n* = 129^a^	p-value^b^
HBV test results				.43
Detectable anti-HBs	303 (91%)	182 (89%)	121 (94%)	
Current infection	19 (5%)	14 (7%)	5 (4%)	
Resolved infection	12 (4%)	9 (4%)	3 (2%)	
Age				**<0.01**
20–35	93 (28%)	31 (15%)	62 (48%)	
36–46	135 (40%)	85 (41%)	50 (39%)	
47+	106 (32%)	89 (43%)	17 (13%)	
Median age (IQR)	40 (35, 49)	44 (38, 51)	36 (31, 40)	**<.01**
Age at time of vaccination				.10
19–32	126 (38%)	75 (37%)	51 (40%)	
33–42	125 (37%)	71 (35%)	54 (42%)	
43+	83 (25%)	59 (29%)	24 (19%)	
Median age at vaccination (IQR)	35 (30, 42)	36 (30, 43)	35 (30, 39)	.14
Sex				.30
Men	44 (13%)	30 (15%)	14 (11%)	
Women	290 (87%)	175 (85%)	115 (89%)	
Body Mass Index				**<.01**
18.6–25.0	114 (34%)	56 (27%)	58 (45%)	
25.1–30.0	120 (36%)	81 (40%)	39 (30%)	
>30.0	100 (30%)	68 (33%)	32 (25%)	
Occupation				.**03**
Physician	70 (21%)	52 (25%)	18 (14%)	
Laboratorian	43 (13%)	26 (13%)	17 (13%)	
Nurse	175 (52%)	96 (47%)	79 (61%)	
Auxiliary medical staff	46 (14%)	31 (15%)	15 (12%)	
Place of work				.40
Hospital	176 (53%)	104 (51%)	72 (56%)	
Polyclinic	158 (47%)	101 (49%)	57 (44%)	
Work experience				**<.01**
<10 years	90 (27%)	22 (11%)	68 (53%)	
10–20 years	153 (46%)	103 (50%)	50 (39%)	
>20 years	91 (27%)	80 (39%)	11 (8%)	
Median length of service (IQR)	15 (9, 22)	19 (14, 25)	9 (4, 14)	**<.01**
HBV vaccination doses				.13
1	22 (7%)	14 (7%)	8 (6%)	
2	70 (21%)	50 (24%)	20 (16%)	
3 (completed series)	242 (72%)	141 (69%)	101 (78%)	

IQR: Interquartile range, HBV: hepatitis B virus.

^a^n (%); Median (interquartile range)

^b^Pearson’s chi-square; Fisher’s Exact Test; Wilcoxon’s Sign Rank Test for Quantitative Variables

Bold is p-value <0.05.

Among the 303 participants with detectable anti-HBs, the geometric mean titer of anti-HBs was significantly higher among participants whose age at the time of vaccination was 19 to 32 years (Table 2) compared to those in older age groups. Nurses and laboratory staff had a higher geometric mean titer than physicians and auxiliary medical staff (58 and 46 versus 20 and 32, respectively, *p* = .03). Employees of polyclinics and medical workers with more than 20 years of experience had lower titers, than inpatient staff (*p* < .01) and participants with less than 20 years of experience (*p* < .01). HCWs with chronic diseases had significantly lower anti-HBs titers than healthy participants (12 vs. 46, *p* < .01). Although the geometric mean titer value was lower in the vaccinated group in 2015 than in those vaccinated in 2022 (35 and 52, respectively), the difference was not statistically significant (*p* = .10).

**Table 2.** Geometric mean titer of anti-HBs associated with hepatitis B vaccination among HCWs vaccinated in 2015 and 2022 (*n* = 303), Tashkent, Uzbekistan.

Characteristics	N	GMT^a^	95% CI	p-value^a^
Age at time of vaccination				.**02**
19–32	111	68	47–98	
33–42	116	30	20–46	
43+	76	31	19–52	
Sex				.07
Men	40	19	9–43	
Women	263	46	35–59	
Body Mass Index				.76
18.6–25.0	103	46	30–71	
25.1–30.0	107	41	26–62	
>30.0	93	36	23–57	
Occupation				.**03**
Physician	66	20	11–36	
Laboratorian	39	46	23–92	
Nurse	155	58	41–80	
Auxiliary medical staff	43	32	17–61	
Place of work				**<.01**
Hospital	157	60	43–84	
Polyclinic	146	27	19–39	
Work experience				**<.01**
<10 years	86	50	31–79	
10–20 years	135	56	39–81	
>20 years	82	20	13–32	
Have chronic diseases^b^				**<.01**
Yes	23	12	5–30	
No	273	46	35–59	
Year of vaccination				.102
2015	182	35	26–48	
2022	121	52	35–78	
Smoking status				.92
Former	8	54	6–461	
Current	26	32	12–86	
Never	269	42	32–54	
Vaccine doses				.55
1	6	24	8–69	
2	25	45	26–78	
3 (completed series)	272	42	32–56	

GMT – geometric mean titer, CI – confidence Interval.

^a^One-sided Kruskal – Wallis analysis of variance.

^b^Chronic kidney disease or diabetes mellitus.

Bold is p-value <0.05.

The prevalence ratio for anti-HBs ≥10 mIU/ml was 0.80 (95% CI 0.68–0.95) for HCW who were 33–42-year-old at the time they got vaccinated compared to those who were 19–32-year-olds at the time (Table 3). The prevalence ratio was 1.33 (95% CI 1.07–1.67) for nurses compared to physicians. Although not statistically different, we observed a lower prevalence of protective immunity among people with chronic diseases than those without (52% vs 72%, respectively). There was no difference between those vaccinated in 2015 and 2022 (70% and 71%, respectively). Without statistical significance, among participants who reported receiving only one HepB dose 59% had anti-HBs ≥10 mIU/ml compared to 70% for two doses and 72% for three doses.

**Table 3.** Factors associated with anti-HBs ≥10 mIU/mL among HCWs vaccinated in 2015 and 2022 (*n* = 303), Tashkent, Uzbekistan.

Characteristics	Total *n* = 303	Anti-HBs ≥10 mIU/mL *n* = 214^a^	PR	95% CI	APR	95% CI	P
Overall	303	214 (71%)					
Age at vaccination							
19–32	111	88 (79%)	Ref		Ref		
33–42	116	74 (64%)	0.80	0.68,0.95	0.95	0.89, 1.01	.13
43+	76	52 (68%)	0.86	0.72,1.03	0.98	0.90, 1.06	.50
Sex							
Men	40	26 (65%)	Ref				
Women	263	188 (71%)	1.10	0.87,1.4			
Body Mass Index							
18.6–25.0	103	71 (69%)	Ref				
25.1–30.0	107	79 (74%)	1.07	0.90,1.27			
>30.0	93	64 (69%)	1.00	0.83,1.21			
Occupation							
Physician	66	38 (58%)	Ref		Ref		
Laboratorian	39	28 (72%)	1.25	0.94,1.66	1.09	0.98, 1.21	.11
Nurse	155	119 (77%)	1.33	1.07,1.67	1.09	0.98, 1.21	.11
Auxiliary medical staff	43	29 (67%)	1.17	0.87,1.57	1.08	0.98, 1.20	.13
Place of work							
Hospital	157	122 (78%)	Ref		Ref		
Polyclinic	146	92 (63%)	0.81	0.70,0.94	0.92	0.87, 0.98	.**01**
Work experience							
<10 years	86	60 (70%)	Ref				
10–20 years	135	106 (79%)	1.13	0.95,1.33			
>20 years	82	48 (59%)	0.84	0.67,1.06			
Vaccination year							
2015	182	128 (70%)	Ref				
2022	121	86 (71%)	1.01	0.87,1.17			
Vaccine doses (self-reported)							
1	22	13 (59%)	Ref		Ref		
2	63	44 (70%)	1.18	0.81,1.73	1.08	0.95, 1.24	.20
3(completed series)	218	157 (72%)	1.22	0.85,1.74	1.11	0.98, 1.25	.10
Chronic diseases^b^							
Yes	23	12 (52%)	Ref		Ref		
No	273	197 (72%)	1.38	0.93,2.06	1.11	1.00, 1.25	.06
Smoking status							
Former	8	6 (75%)	Ref				
Current	26	17 (65%)	0.87	0.54,1.42			
Never	269	191 (71%)	0.95	0.63,1.42			
Alcohol use							
Former	6	3 (50%)	Ref				
Current	25	18 (72%)	1.44	0.62,3.32			
Never	272	193 (71%)	1.42	0.64,3.17			

PR – Unadjusted Prevalence Ratio, APR – Adjusted prevalence ratio.

^a^n (%).

^b^Chronic kidney disease and diabetes mellitus included.

Bold is p-value <0.05.

In a multivariable model, which adjusted for age, presence of chronic disease and number of vaccination doses, HCW who worked in polyclinics had 8% decreased prevalence of protective immunity compared to those that worked in hospitals.

In bivariable analysis of factors associated with successful completion of the 3-dose HBV vaccine series, only place of work was significant (Table 4). HCW who worked in polyclinics were more likely to have completed the 3-dose series than those who worked in hospitals (76% vs 66%, *p* = .04)

**Table 4.** Factors associated with self-reported completion of the 3-dose HBV vaccination series among HCWs vaccinated in 2015 and 2022 (*N* = 303), Tashkent, Uzbekistan.

Characteristic	Did not, *N* = 85^a^	Completed 3 doses, *N* = 218^a^	p-value^b^
Age at time of vaccination			.20
19–32	31 (28%)	80 (72%)	
33–42	38 (33%)	78 (67%)	
43+	16 (21%)	60 (79%)	
Sex			.40
Men	9 (23%)	31 (78%)	
Women	76 (29%)	187 (71%)	
Occupation			.30
Physician	15 (23%)	51 (77%)	
Laboratorian	8 (21%)	31 (79%)	
Nurse	47 (30%)	108 (70%)	
Auxiliary medical staff	15 (35%)	28 (65%)	
Place of work			.**04**
Hospital	52 (33%)	105 (67%)	
Polyclinic	33 (23%)	113 (77%)	
Work experience			.50
<10 years	24 (28%)	62 (72%)	
10–20 years	42 (31%)	93 (69%)	
>20 years	19 (23%)	63 (77%)	
Year of vaccination			.07
2015	58 (32%)	124 (68%)	
2022	27 (22%)	94 (78%)	
Protective immunity	57 (27%)	157 (73%)	.40

^a^n (%).

^b^Pearson’s Chi-squared test.

Bold is p-value <0.05.

## Discussion

We assessed prevalence of anti-HBs ≥10 mIU/ml in HCW vaccinated during the hepatitis B vaccination campaigns in 2015 and 2022 and found that 5% had an active HBV infection, 4% had a resolved infection, and 91% had detectable anti-HBs levels. Among these, only 71% had protective levels of anti-HBs ≥10 mIU/ml. Anti-HBs ≥10 mIU/ml prevalence measured one month after completion of vaccination is estimated to be 92% among HCW <40 years old and 84% among those ≥40 years old.^18,19^

Although anti-HBs levels can be detected more than 30 years after vaccination,^20^ they decrease over time and can drop below 10 mIU/ml, especially for people who were poor or moderate responders after vaccination (<1000 mIU/mL).^21^ Because anti-HBs titers are known to wane over time,^22,23^ we had expected that the prevalence of seroprotection would have been low overall and lower among HCW who were vaccinated in 2015 than those vaccinated in 2022. But we found no significant difference in the prevalence of participants with anti-HBs ≥10 mIU/ml by vaccination year. We also didn’t observe a statistically significant difference in the mean geometric titers of HCW vaccinated in 2015 compared to those vaccinated in 2022.

The prevalence of HCW with below protective titers of 29% in our study, differs from studies in different populations in Uzbekistan, which found that 18% of children had low anti-HBs levels five years after vaccination.^24^ But when comparing our prevalence with similar studies among HCW, our findings are consistent.^25,26^ For example, a cross-sectional study of HCW in Uganda found 72% of HCW vaccinated up to 10 years prior still had anti-HBs ≥10 mIU/ml.^27^

We found low compliance with the 3-dose vaccination series and 28% of workers did not complete the entire three-dose series. Though not statistically significant, the proportion with protective levels of anti-HBs antibodies was lower for those with one dose than those who completed the 3-dose series. The lack of statistical significance might be because of the lack of power to detect differences in small subgroups, as this was not the primary objective of our study. Healthcare workers who have not completed all 3-doses of the vaccination series are considered to be incompletely vaccinated and are at risk for Hepatitis B exposure.^28^ These workers are at risk for Hepatitis B infection. Revaccination with the full 3-doses followed by anti-HBs testing 1–2 months after the final vaccine dose of healthcare workers that lack documentation of the vaccine doses they received and completion of the missing doses from the 3-dose series for those with valid documentation of completed doses is important to ensure they are protected.^8,9^ Nearly 6,000 healthcare workers in Uzbekistan may be incompletely vaccinated if 28% of the 20,600 healthcare workers vaccinated during the two campaigns did not complete the 3-doses. In bivariable analysis, the proportion with anti-HBs ≥10 mIU/ml was significantly higher among laboratory staff and nurses than among physicians. Only half of physicians in the study developed protective immunity. Given the small number of physicians included in this study, there was not sufficient power to do subgroup analysis to understand specific risks for this population. Additional studies are needed to further understand if this is a broader issue in Uzbekistan beyond this study.

Uzbekistan does not have a policy for serological testing of HCW after completion of the hepatitis B vaccination 3-dose series, nor a policy for revaccination of workers who are non-responders to the first 3-dose series and do not have current infection. Based on the proportion of HCW with HBs < 10 mIU/ml, about 5,500 hCW vaccinated in the two campaigns may not be fully protected against Hepatitis B. Studies have found that 44–100% of people who fail to form protective titers to an initial series go on to develop protective titers to a 3-dose revaccination series.^29,30^ Therefore, the adoption of post-vaccination testing with revaccination when needed could help ensure that HCW are protected from hepatitis B in Uzbekistan where an estimated (8.3%) 2.5 million people have HBV.^12^ In addition, 5.6% of HCWs in our study were shown to have current HBV infection. Referral to care and treatment for HCWs who test positive for HBsAg and implementation of infection prevention and control measures would prevent further infections in healthcare settings.^13,31^

The results of our study are subject to some limitations. First, we relied on self-reported vaccination history data, including data on the number of doses and vaccination intervals. We were able to verify dose numbers for 11% of participants, who provided access to their vaccine records. We found 100% concordance between self-reported and documented vaccination histories. Second, our study was conducted at people’s place of work. This might have resulted in social desirability bias wherein participants would have underreported stigmatized risk factors such as smoking or drinking and over reported number of vaccine doses (for participants that did not have documented records). Third, serological testing was conducted up to 8 years after vaccination, and therefore does not reflect post-vaccination anti-HBs response and limits comparability with other studies that conduct testing one-month after completion of the vaccine series. Fourth, the study was not powered to detect differences between small groups and within subgroups, for example we lacked the power to do subgroup analysis for each occupational group. Similarly, we had insufficient power to test the difference between HCW who only received one dose versus two or three doses because only six participants were in the one dose group. Lastly, our results are limited to Tashkent, where vaccination was performed, and cannot be extrapolated to other cities of Uzbekistan.

Our study was the first study in Uzbekistan to assess anti-HBs levels among vaccinated HCW. We found that 71% of HCW vaccinated against hepatitis B in 2015–2022 had protective anti-HBs ≥10 mIU/ml in 2023. Adoption of post-vaccination serological testing can help identify HCW who fail to develop a protective immune response that requires revaccination and refer to care and treatment HCWs who are diagnosed with chronic HBV infection. Strategies to increase compliance with the recommended vaccination schedule are important to ensure protection in this population at high risk of occupational exposure to HBV and ensure patient safety.

## Data Availability

The datasets used and/or analyzed during the current study are available from the corresponding author on reasonable request.
